# Biological Microbial Interactions from Cooccurrence Networks in a High Mountain Lacustrine District

**DOI:** 10.1128/msphere.00918-21

**Published:** 2022-06-01

**Authors:** Vicente J. Ontiveros, Rüdiger Ortiz-Álvarez, José A. Capitán, Albert Barberán, David Alonso, Emilio O. Casamayor

**Affiliations:** a Integrative Freshwater Ecology, Centre for Advanced Studies of Blanes, Spanish Research Council (CSIC), Blanes, Spain; b Theoretical and Computational Ecology, Centre for Advanced Studies of Blanes, Spanish Research Council (CSIC), Blanes, Spain; c Complex Systems Group, Department of Applied Mathematics, Universidad Politécnica de Madrid, Madrid, Spain; d Department of Environmental Science, University of Arizona, Tucson, Arizona, USA; University of Michigan—Ann Arbor

**Keywords:** cell-cell interaction, microbes, networks

## Abstract

A fundamental question in biology is why some species tend to occur together in the same locations, while others are never observed coexisting. This question becomes particularly relevant for microorganisms thriving in the highly diluted waters of high mountain lakes, where biotic interactions might be required to make the most of an extreme environment. We studied a high-throughput gene data set of alpine lakes (>220 Pyrenean lakes) with cooccurrence network analysis to infer potential biotic interactions, using the combination of a probabilistic method for determining significant cooccurrences and coexclusions between pairs of species and a conceptual framework for classifying the nature of the observed cooccurrences and coexclusions. This computational approach (i) determined and quantified the importance of environmental variables and spatial distribution and (ii) defined potential interacting microbial assemblages. We determined the properties and relationships between these assemblages by examining node properties at the taxonomic level, indicating associations with their potential habitat sources (i.e., aquatic versus terrestrial) and their functional strategies (i.e., parasitic versus mixotrophic). Environmental variables explained fewer pairs in bacteria than in microbial eukaryotes for the alpine data set, with pH alone explaining the highest proportion of bacterial pairs. Nutrient composition was also relevant for explaining association pairs, particularly in microeukaryotes. We identified a reduced subset of pairs with the highest probability of species interactions (“interacting guilds”) that significantly reached higher occupancies and lower mean relative abundances in agreement with the carrying capacity hypothesis. The interacting bacterial guilds could be more related to habitat and microdispersal processes (i.e., aquatic versus soil microbes), whereas for microeukaryotes trophic roles (osmotrophs, mixotrophs, and parasitics) could potentially play a major role. Overall, our approach may add helpful information to guide further efforts for a mechanistic understanding of microbial interactions *in situ*.

**IMPORTANCE** A fundamental question in biology is why some species tend to occur together in the same locations, while others are never observed to coexist. This question becomes particularly relevant for microorganisms thriving in the highly diluted waters of high mountain lakes, in which biotic interactions might be required to make the most of an extreme environment. Microbial metacommunities are too often only studied in terms of their environmental niches and geographic barriers since they show inherent difficulties to quantify biological interactions and their role as drivers of ecosystem functioning. Our study highlights that telling apart potential interactions from both environmental and geographic niches may help for the initial characterization of organisms with similar ecologies in a large scope of ecosystems, even when information about actual interactions is partial and limited. The multilayered statistical approach carried out here offers the possibility of going beyond taxonomy to understand microbiological behavior *in situ*.

## INTRODUCTION

The occurrence of specific microbes in natural environments is associated with multiple processes. At the spatial scale, geographic distance may create an effect of dispersal limitation (1), but most importantly, the local environment exerts strong filters facilitating the settlement of microbes with similar environmental preferences (2). A good example is the effect of pH on freshwater ecosystems, where a regional pool of microbes distributed along the hydrographical network is filtered according to water alkalinity, among other environmental reasons (3, 4). Although the environment has been extensively studied under the lens of community ecology as a driver of community assembly (4, 5), environmental factors or even dispersal limitation can be incomplete predictors of joint microbe occurrence (6). There is often a lack of understanding about the direct interrelationships among microbes—i.e., biological and ecological interactions—and how these are crucial in regulating the appearance or abundance of species in complex communities (7, 8). Unfortunately, there is a lack of knowledge about the myriad of interactions (9, 10) or ecological roles (9, 11, 12) of many community members. Microbial ecology has traditionally studied microbial interactions by analyzing small sets of species in controlled environments such as in cocultures (13) and biofilms (14), as well as population-level responses through quorum sensing (15) or particular interactions such as competition (7). For example, this issue was easily observed in microeukaryotes under a microscope, spotting the parasitic behavior of aquatic fungi toward phytoplankton (16) or the predatory effect of zooplankton toward phytoplankton (17). Still, there are potentially many specific and unspecific interactions that have not yet been detected but that may be particularly relevant in organizing biological communities. So, when direct observation of interactions is difficult, ecologists may rely on computational methods to infer potential interactions. For example, signals of competitive interactions have been suggested in the case of macroorganisms (18).

Recently, amplicon sequencing through high-throughput technologies has allowed microbial ecologists to study multiple species thriving in their natural communities. Through the inference of cooccurrences and coexclusions, many studies have attempted to unveil why some species tend to occur together in the same locations, while others are never observed sharing the same ecological space (19–21). These associations and network properties linked to the association’s structure have been traditionally hard to interpret, since occurrences based on spatial associations do not solely account for biotic interactions and usually blend patterns of interactions and environment (22, 23). To decipher the causes acting on associations (cooccurrences and coexclusions) and infer potential biotic interactions, the study of microbial associations should be conducted within a defined metacommunity (24), i.e., a set of local communities, linked by dispersal, of potentially interacting species (25). A good model system would be one where local communities have some degree of isolation to display specific environmental filters but still are connected enough to be part of the same metacommunity. For this reason, in this study, we used a high mountain lacustrine district (Pyrenees, Spain) with 224 sampled lakes (local communities) comprising a regional metacommunity. In lacustrine districts, each lake acts as a local community, isolated by land but closely influenced by regional processes such as atmospheric deposition (26), catchment processes (27), and water flows (3), all mobilizing the regional species pool (28). Although interactions occur anywhere and can be extremely varied, in habitats with a strong nutrient limitation, such as in the highly diluted waters of high mountain lakes, food-web components and functional interactions are the microbial engine responsible for nutrient mobilization (29–32).

The purpose of this study was 2-fold. First, to quantify the drivers of species associations in a high-altitude lacustrine microbial metacommunity, and second, to define potential microbial interaction units and explore their properties. To do so, we identified microbial associations caused by both environmental niches and dispersal limitation, interpreting the unexplained associations as potential biotic interactions. This approach allowed us to extract several layers of information, starting by describing the environmental variables that were primarily linked to taxon associations and ranking the influence of these factors to explain cooccurrence and coexclusion pairs. Once the importance of explanatory variables on association pairs had been quantified, we examined specifically the pairs interpreted as species interactions. By integrating pairs into a network analysis, we sought to describe different groups of species interactions on the basis of network properties and modules. We classified taxa into clusters of more-positive associations and less-negative associations to find groups of species based on interactions (“interacting guilds”) and keystone taxa organizing each group. Targeting these keystones and their guilds in future studies adds a powerful new layer of information based on interactions to the traditional approaches based on taxonomy or functional components in tag-sequencing studies.

For this purpose, we applied the combination of a probabilistic method for estimating significant cooccurrences/exclusions and a conceptual framework for filtering out associations potentially linked to environmental and/or spatial factors.

## RESULTS AND DISCUSSION

In the present study, we applied a combination of tools to tell apart biotic interactions from associations driven by niche differentiation or geographic dispersal limitation, using a large amplicon high-throughput sequencing data set. We first inferred significant associations by combining a probabilistic method, followed by variance analysis, to separate the species pairs that can be explained by environmental variables or geographic coordinates. This computational method allowed us to rank the influence of environmental factors and to identify the pairs interpreted as species interactions. By integrating these pairs into a network analysis, we described different groups of species interactions on the basis of network properties and modules, as well as keystone taxa organizing each group. Targeting these unseen keystones and their guilds added a new view based on interactions to the traditional approaches mostly based on taxonomy or functional components in tag-sequencing studies.

### Quantifying environmental niches and isolating potential biotic interactions.

**FIG 1:**
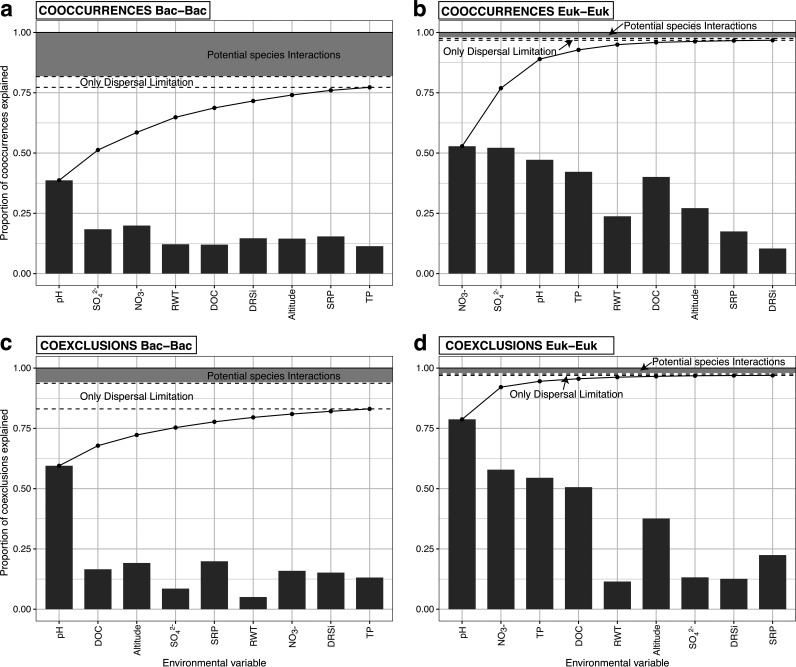
Proportions of cooccurrence and coexclusion pairs (*Bacteria*-*Bacteria* [a and c] and *Eukarya*-*Eukarya* [b and d]) estimated using the probabilistic approach, explained by the environment and the geography after conducting ANOVA and MANOVA tests, respectively. Environmental variables were ranked based on the cumulative proportion of links not previously explained by any other environmental variables (black line).

The applied approach identified microbial associations caused by both environmental niches and dispersal limitation, interpreting the unexplained associations as potential biotic interactions. The probabilistic method for the calculation of co-occurrences retrieved a total of 52,143 significant cooccurrences and 25,675 coexclusions, of 661 bacterial and 265 eukaryotic zero-radius operational taxonomic units (zOTUs) with 427,812 potential combinations. These pairs harbor significant associations primarily reflecting environmental (analysis of variance [ANOVA] test: 83.34% of pairs) and dispersal (multivariate ANOVA [MANOVA] test: 4.14% of unique pairs) processes. Indeed, cooccurrences have been often used before to show the ecological clusters based on environmentally driven edges (33). Hence, we quantified the environmentally driven edges by sorting the environmental variables based on the unique proportion of cooccurrences and coexclusions explained for each variable. The analysis showed distinct rankings for bacteria and microbial eukaryotes (Fig. 1). In general, environmental variables explained fewer pairs in bacteria than in microbial eukaryotes, with pH alone explaining the highest proportion of bacterial pairs, and leaving 8,202 unexplained pairs by any variable (Fig. 1a and c). Dispersal limitation alone, stood out in explaining ~10% of bacterial coexclusions but did not explain a high proportion of the cooccurrences (~2%). In *Eukarya*, cooccurrences were mostly explained by NO_3_^–^ and SO_4_^2+^ (Fig. 1b) and coexclusions by pH (Fig. 1d). Interestingly, most eukaryotic pairs were explained by environmental and geographic variables, leaving only 212 pairs unexplained. With regard to the unexplained pairs, there are examples in the literature where these precise associations have been explored to search for ecological interactions at the community and regional levels (34, 35), which highlights the potential of the approach. In total, our analyses left 9,749 pairs unexplained (12.52% of the total, 7,017 cooccurring, 2,732 coexclusions), which may be interpreted as potential biotic interactions.

### Explanatory variables of associated pairs according to their taxonomic rank.

**FIG 2:**
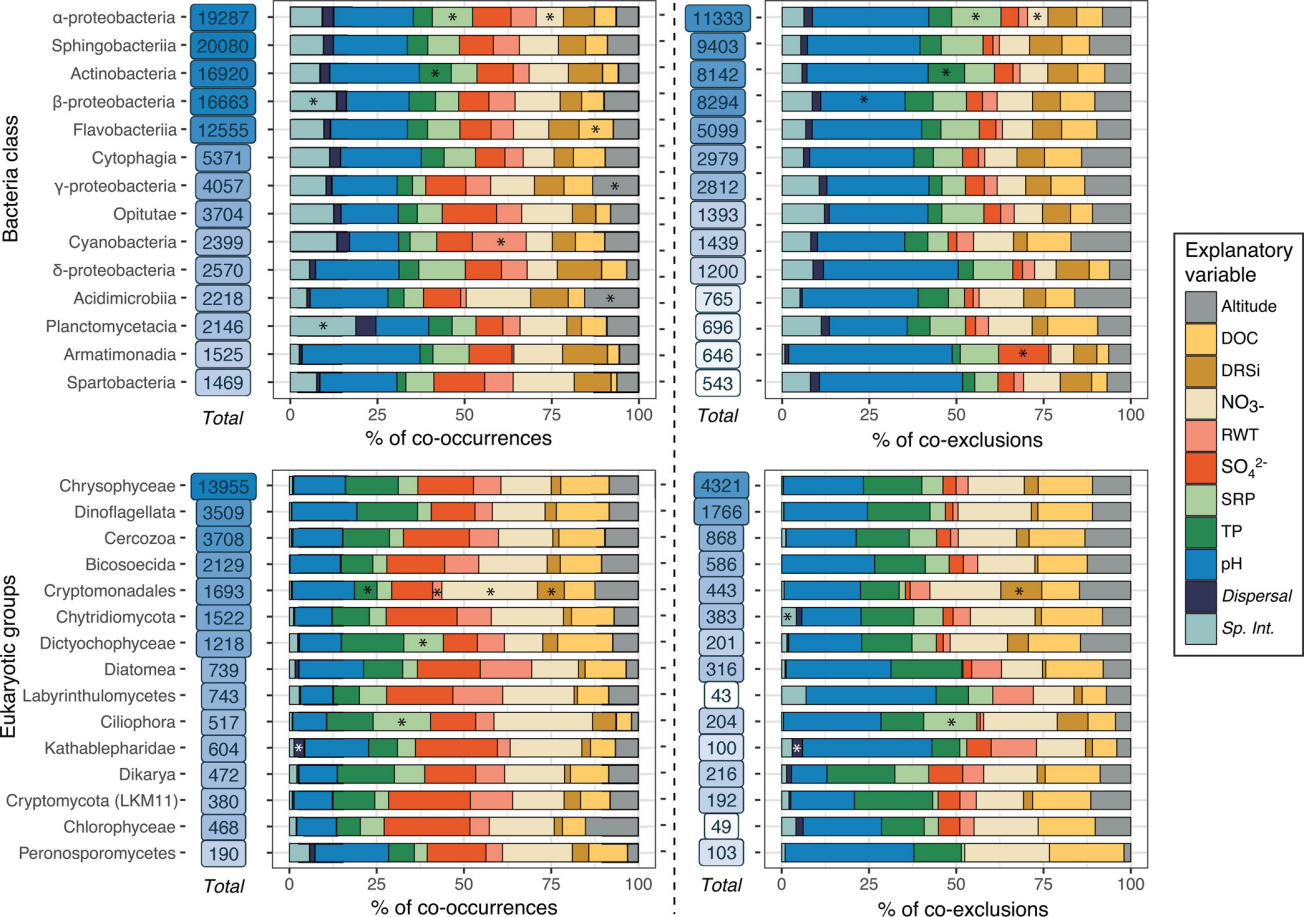
Proportions of variables (environment, dispersal, or potential species interaction) explaining cooccurrences and coexclusions across taxonomic groups. A single pair constitutes two nodes with the same or different taxonomy; hence, a pair could contribute to two different taxa. The bar plot displays the dominant bacterial classes and ecologically relevant eukaryotic groups, and the number of pairs by taxa subject to an explanatory variable. The asterisks (*) indicate the highest contributors to the significant association between bar and explanatory variables according to a chi-square test.

When combining explanatory variables with taxonomy there was an apparent equilibrium at high taxonomic ranks (i.e., class), with taxa similarly explained by the different variables (Fig. 2). However, there were higher differences between bacterial groups at the genus level (see Fig. S1 and Table S1 in the supplemental material). We did not narrow down to the genus level in eukaryotes because of classification limitations based on the short ribosomal region used (V9, in the 18S-rRNA) (36). In all the cases, we used a chi-square test of independence to analyze the contingency table of the absolute associations between explanatory variables and microbial taxa to explore significant associations (*P* < 0.0001). The pH was the variable explaining more associations both in *Bacteria* and in *Eukarya* and promoted the networks with the highest clustering coefficients. It has been shown that pH is one of the strongest predictors of microbial communities in natural ecosystems such as soils (33, 37), and freshwater ecosystems (3, 4). However, there were no differences regarding how pH explained associations for each high-rank taxon, except for a lower proportion of coexclusions involving *Betaproteobacteria*. In addition, narrowing down at bacterial genus level (see Table S1), there were PAM k-medoids clusters with more pairs (K2) explained by pH, or with less pairs (K3), showing in turn an increase of pairs explained by nutrient variables (DOC, NO_3_^–^, and total phosphorous [TP]). Indeed, nutrients were the second most important group of variables explaining association pairs, particularly in *Eukarya*.

10.1128/msphere.00918-21.2 FIG S1 (a) Bar plot displaying the proportions of variables (environment, dispersal, or potential species interaction) that explain cooccurrences and coexclusions across taxonomic bacterial genera, according to the results obtained using the method of Blois et al. (22). A single pair constitutes two nodes with the same or different taxonomy; hence, a pair could contribute to two different taxa. The bar plot displays the groups of genera according to a partitioning around medoids clustering, relying on the proportion of explanatory variables over the pairs where each genus was participating. (b) Contribution in percentage of the Pearson residuals to the total chi-square value of cooccurrences (left) and coexclusions (right). Download FIG S1, PDF file, 0.2 MB. Copyright © 2022 Ontiveros et al. 2022 Ontiveros et al. https://creativecommons.org/licenses/by/4.0/ This content is distributed under the terms of the Creative Commons Attribution 4.0 International license.

10.1128/msphere.00918-21.7 TABLE S1 Bacterial genera grouped according to PAM k-medoids clustering. Download Table S1, DOCX file, 0.01 MB. Copyright © 2022 Ontiveros et al. 2022 Ontiveros et al. https://creativecommons.org/licenses/by/4.0/ This content is distributed under the terms of the Creative Commons Attribution 4.0 International license.

Although small differences were observed in *Bacteria*, it is worth to mention that in *Flavobacteria* cooccurrences, dissolved organic carbon (DOC) explained a higher proportion of pairs, agreeing with previous findings where flavobacterium-like populations were favored during periods of high heterotrophic activity with a high availability of resources (38). Sulfate was also an important explanatory variable of association pairs, particularly in eukaryotic cooccurrences, but scarcely in their coexclusions. Despite not explaining a lot of pairs in *Bacteria*, there were more pairs explained by sulfate when the pair involved *Armatimonadia* or the K7 cluster, than in other taxa.

Other variables that to a lower extent may form niches are water-renewal time (RWT), explaining more cyanobacterial pairs, SRP, explaining more *Dictyochophyceae* and *Ciliophora* associations, and the K4 PAM k-medoids bacterial cluster, and DRSi, explaining more *Cryptomonadales* associations. Altitude, as a proxy of temperature and UV radiation, seemed to explain more pairs involving *Alphaproteobacteria* and *Acidimicrobia* than in other taxa.

**FIG 3:**
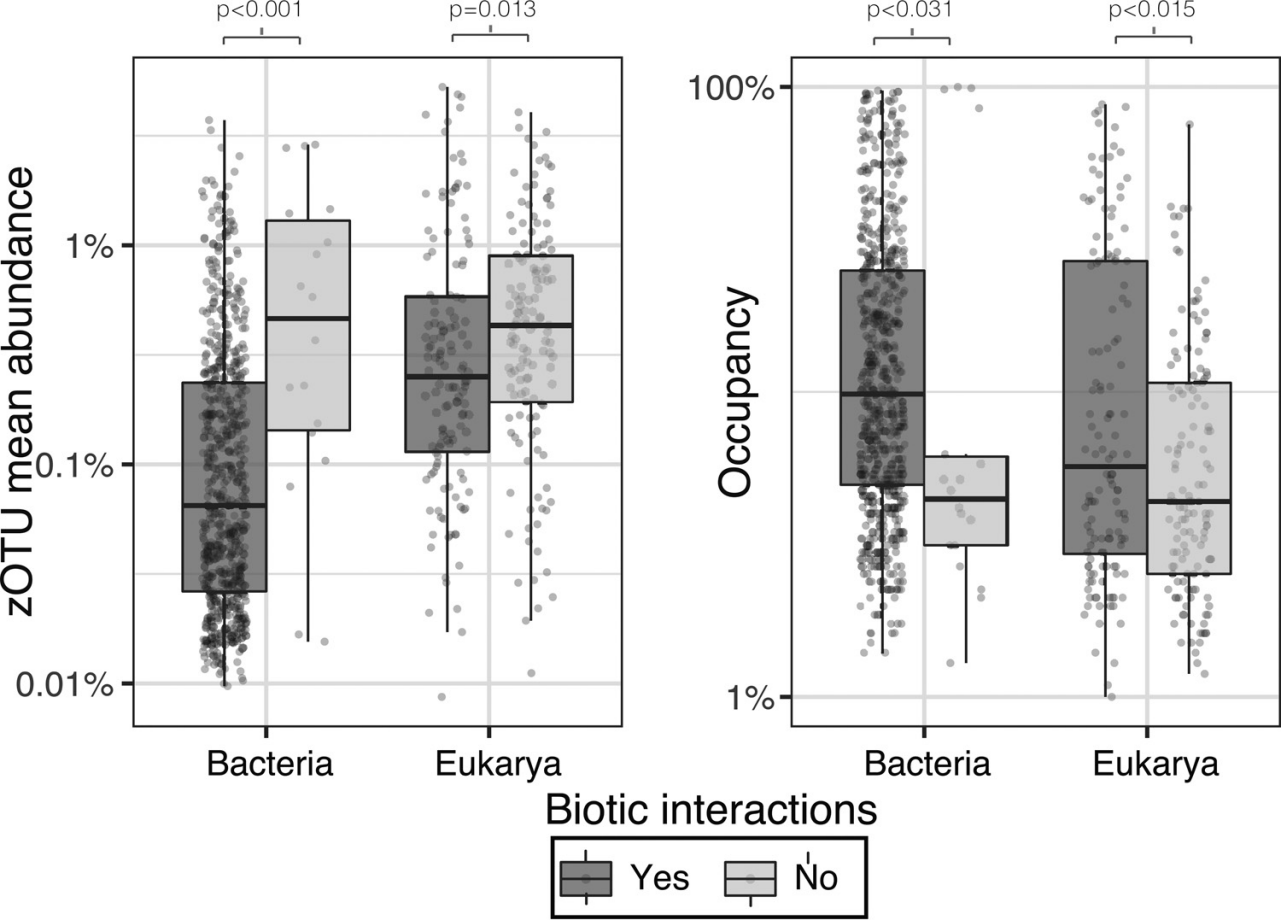
Mean regional relative abundances and regional occupancy between interacting zOTUs or noninteracting zOTUs in bacterial and microbial eukaryotes.

Assuming that we covered the most important environmental variables, as we had shown in previous studies using the same data set (4, 39), we argue that the remaining pairs could be interpreted as potential positive and negative biotic interactions, which are differently quantified and balanced depending on the taxonomic affiliation (see Fig. S2). Although we cannot rule out unmeasured environmental variables or other factors explaining some pairs, the method selected a reduced subset of pairs with the highest probability of species interactions. In fact, microbes that potentially interact, significantly reached higher occupancies than those that do not interact (*Bacteria*: *P* < 0.031, *Eukarya*: *P* < 0.015) (Fig. 3, right panel). Most importantly, there were significant abundance differences between those microbes that potentially interact (with lower mean relative abundances) and those that do not interact (Wilcox test, *Bacteria*: *P* < 0.001, *Eukarya*: *P* < 0.013). Indeed, this result has previously been observed in protist experiments (40) and has been explained by the carrying capacity hypothesis (41), in which the maximal abundance is given by the system resources and its idiosyncrasies. Also, microbes that cooperate with many other microbes need to share the available resources while limiting their abundances. This trend reflects the previous observation that niche-expanding positive nontrophic interactions (i.e., facilitation, mutualism, or commensalism) (42) seem to be overrepresented in the pool of inferred potential interactions (43).

10.1128/msphere.00918-21.3 FIG S2 Summary of estimated interactions by taxonomic groups in *Bacteria* (a) and *Eukarya* (b), and interdomain (c). Raw counts, without weights. (a) In terms of taxonomic groups, in *Bacteria*, members of *Burkholderiales* (*Betaproteobacteria*), *Sphingobacteriales*, *Flavobacterales*, and *Cytophagales* (*Bacteroidetes*), *Caulobacterales* (*Alphaproteobacteria*), and *Frankiales* (*Actinobacteria*) held the most interactions. (b) Except the orders *Pseudomonadales* (*Gammaproteobacteria*) and *Oligoflexales* (Deltaproteobacteria), all orders had more positive than negative potential interactions. In the case of *Eukarya*, most potential interactions were quantified in the dominant group *Chrysophyceae*, followed by *Chytridiomycota*, *Dictyochophyceae*, *Cercozoa*, and *Dinoflagellata*. Only *Cryptomycota* had more negative than positive potential interactions, and *Tracheophyta* only was involved in negative pairs. (c) Bacterial relationships with eukaryotes were concentrated in *Chrysophyceae* and then in *Dinoflagellata*, *Chytridiomycota*, and *Cercozoa*. Interestingly, 9 groups (of 17) had more negative than positive interactions: *Tracheophyta*, *Chlorophyceae*, *Bicosoecida*, *Cryptomycota*, *Dikarya*, *Cryptomonadales*, *Dictyochophyceae*, *Peronosporomycetes*, and *Cryptomycota*. Download FIG S2, PDF file, 0.1 MB. Copyright © 2022 Ontiveros et al. 2022 Ontiveros et al. https://creativecommons.org/licenses/by/4.0/ This content is distributed under the terms of the Creative Commons Attribution 4.0 International license.

Overall, after separating the cooccurrences and coexclusions that could not be explained by overlapping environmental niches or geographic dispersal, the resulting networks were closer to an ecological network rather than a simple associational network, integrating multiple types and effects of ecological interactions, direct and indirect, taking place across spatial scales from regional to local. In these networks, the internal ecological structure should harbor subgroups of organisms based on an ecological criterion. Although there is some ecological coherence in high-rank bacterial taxa (44), defining alternative groups with a common ecology rather than those with a common phylogeny (45) may be more relevant in terms of biological interactions.

We arranged positive and negative potential biological interactions into domain networks that were subsequently divided in three modules both in bacteria (B1, B2, and B3, respectively, spinglass modularity = 0.31) and in eukaryotes (E1, E2, and E3, respectively, spinglass modularity = 0.53). Using modularity to “separate” subcommunities within a metacommunity network is a common strategy (46–48), often assumed to divide a metacommunity network into theoretical community types that would be naturally found in the local samples. These modules hold structural characteristics that can be objectively measured such as diameter, clustering coefficient, average path length, and the average hub value of their nodes (Table 1), weighted degree distributions (see Fig. S3), between-module arrangements (see Fig. S4), or properties quantified at the local community level such as richness and abundance per lake, among others (see Fig. S5). These modules promote within-module cooccurrences, and intermodule coexclusions. These groups can be considered “interacting guilds,” according to a very broad definition: “a group of species that are similar in somewhat that is ecologically relevant, or might be” (45). Since there is not a single definition of “guild,” the criterion we use here to characterize guilds was to separate groups of microorganisms that potentially interact more than expected by chance.

**TABLE 1:** Properties of network modules^*a*^

Module	No. of nodes	Diam	Clustering	Avg path length	Mean Hub value (SD)
B1	268	26.93	0.10	2.81	0.01 (0.02)
B2	186	23.10	0.19	2.54	0.05 (0.09)
B3	189	18.35	0.35	2.13	0.18 (0.19)
E1	54	15.23	0.38	1.63	0.01 (0.02)
E2	52	42.67	0.04	3.38	0.02 (0.13)
E3	26	68.46	0.12	5.54	0.00 (0.00)

a In eukaryotes, properties exclude five unconnected pairs. The number of edges and the distribution within and between modules is presented in detail in Fig. S4. Additional module metrics are available in Fig. S5.

10.1128/msphere.00918-21.4 FIG S3 Density distribution of node weighted degree, displayed for the modules in the network of bacteria (a) and in the network of eukaryotes (b). The mean value of nodes from each module was drawn with a dashed line. Download FIG S3, PDF file, 0.1 MB. Copyright © 2022 Ontiveros et al. 2022 Ontiveros et al. https://creativecommons.org/licenses/by/4.0/ This content is distributed under the terms of the Creative Commons Attribution 4.0 International license.

10.1128/msphere.00918-21.5 FIG S4 Number of edges between and within modules of the *Bacteria* network (B1, B2, and B3) and the *Eukarya* network (E1, E2, and E3) and interdomain relationships based on domain-specific modules. Download FIG S4, PDF file, 0.1 MB. Copyright © 2022 Ontiveros et al. 2022 Ontiveros et al. https://creativecommons.org/licenses/by/4.0/ This content is distributed under the terms of the Creative Commons Attribution 4.0 International license.

10.1128/msphere.00918-21.6 FIG S5 Local community quantification of network metrics: module richness/lake (number of zOTUs within a module that are present in each individual sample), module completeness/lake (proportion of nodes from each module that is present in each individual sample, regarding the total number of nodes of each module), module abundance/lake (the total relative abundance of the nodes corresponding to each module), and total node weight/lake (sum of node weighted degree by each module and each lake). Download FIG S5, PDF file, 0.1 MB. Copyright © 2022 Ontiveros et al. 2022 Ontiveros et al. https://creativecommons.org/licenses/by/4.0/ This content is distributed under the terms of the Creative Commons Attribution 4.0 International license.

### Understanding interaction guild components and keystone taxa.

**FIG 4:**
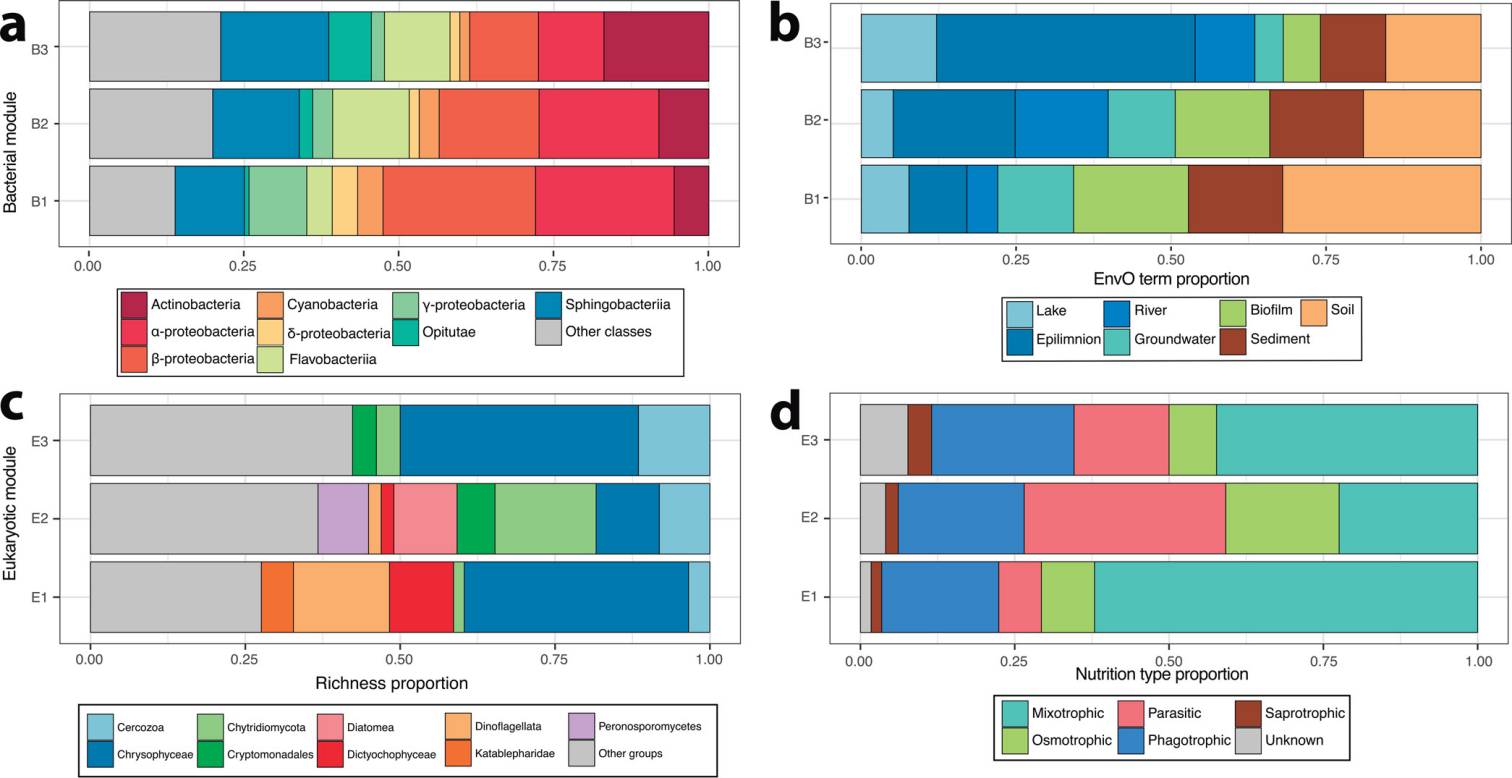
Bar plots showing the relative abundances of bacterial taxonomic groups (a) and bacterial associated EnvO terms (b) and the relative abundances of eukaryotic taxonomic groups (c) and nutrition strategies (d) for the different biotic-driven modules (“interacting guilds”) found.

A look in detail for Bacteria showed different properties in the three guilds (Fig. 4a and b; see also Fig. S3 and S5): (i) B1, a low-abundance module with a degree distribution skewed toward negative values, dominated by *Alpha*-, *Beta*-, and *Gammaproteobacteria*, from soil, sediment, and biofilm habitats; (ii) B3, a high-abundance module with a degree distribution skewed toward positive values, dominated by *Actinobacteria*, *Sphingobacteria*, and *Opitutae*, from aquatic habitats; and (iii) B2, with intermediate characteristics between B1 and B3. Interestingly, according to the environmental ontology annotations of their nodes, the interacting bacterial guilds may be related to habitat and microdispersal processes (i.e., aquatic versus soil microbes). This phenomenon has been recognized as an important structuring factor in freshwater ecosystems (49). In the case of *Actinobacteria* from module B3, it could be a good example of a potential keystone taxa that deserves further investigations. *Actinobacteria* have actinorhodopsins (50, 51), which may function as energy generation for the trophic chain in ultraoligotrophic alpine lakes. Hypothetically, *Actinobacteria* may act in these systems as a central community component where other organisms may arrange around to form an interacting guild.

In the case of *Eukarya*, the interacting guilds were split into three categories, as follows: (i) E1, high-abundance mixotrophs with high total weights and an extended degree distribution; (ii) E2, low-abundance parasites and osmotrophs such as *Peronosporomycetes*, *Chytridiomycota*, and *Diatomea*, exhibiting a peaked degree distribution skewed toward negative degrees; and (iii) E3, showing the lowest abundance and total node weights, characterized by *Cercozoa* and *Chrysophyceae*, and displaying a degree distribution skewed toward positive values (Fig. 4c and D; see also Fig. S3 and S5). Each of these guilds has taxa whose ecologies are related to their interactions, and module E2 (fungal parasites) might be particularly relevant. The role of fungi in freshwater ecosystems has been highlighted as a modulator of dynamics by releasing nutrients through the degradation of organic matter, although their roles are still being resolved (9, 16). Given that module E2 has most negative interactions with modules E1 and E3 (phytoplankton) but also curiously harbors positive interactions with *Diatomea*, we wonder whether diatoms can properly manage nonlethal highly specific infections by parasitic/saprotrophic fungi in high mountain lakes, a question meriting further study. Overall, these results strongly suggest that the unveiled bacterial and eukaryotic interacting guilds found have differences based on ecological categories.

### Limitations and opportunities of the approach.

We carried out an approach that certainly has the potential to tease apart the influence of the environment and dispersal limitation on community assembly in microbial systems but that also has some limitations that must be acknowledged to avoid interpretations based on false premises. Methodologically, we dealt with presence-absence data and not real abundances. This limitation may obscure the interaction-abundance relationships, but presence-absence has the advantage that it may detect nonlinear relationships that pure correlation methods cannot detect. In addition, there might be other ecological processes that drive joint species occurrence, such as particular dispersal mechanisms taking place in freshwater environments, without actually interacting. For instance, habitat dispersal at the catchment scale in the bacterial component (4, 49) is not easy to isolate, and may add noise to the filtered potential interactions. Also, we might have missed relevant environmental variables that could explain some of the species pairing. However, it seems unlikely that new variables could explain a significant portion of the potential interactions, since additional variables incurred in decreasing percentages of previously unexplained pairs (Fig. 1) and the selected variables already represent the environmental variation at a regional scale. Despite these uncertainties, unexplained pairs are good candidates to correspond to actual biotic interactions and may add helpful information to guide further efforts for a mechanistic understanding of microbial interactions *in situ*. By narrowing down potential biological interactions with association networks there is the possibility of discovering new interactions that were missed by traditional methods. In our study, we highlight the association networks with which taxa are most likely to establish interactions.

### Concluding remarks.

We unveiled here potential keystone taxa for the defined interacting units and showed that even phylogenetically related organisms behave very differently in terms of abundance and interaction strength according to their assigned role. Understanding the interaction of organisms within these metacommunities is the basis for unveiling the processes behind ecological organization. Microbial metacommunities are too often only studied in terms of their environmental niches and geographic barriers due to inherent difficulties that make it harder to quantify biological interactions and their role as drivers of ecosystem functioning. Our study highlights that distinguishing potential interactions in both environmental and geographic niches may help in the initial characterization of organisms with similar ecologies in a large scope of ecosystems, even when information about actual interactions is partial and limited. The multilayered statistical approach carried out here offers the possibility of going beyond taxonomy to understand microbiological behavior *in situ*.

## MATERIALS AND METHODS

### Data sets and metadata.

The metacommunity was composed of lake surface plankton samples that were taken during summer of 2011 along the Pyrenees mountain range (4). Most of these lakes lie within ultraoligotrophic/oligotrophic ranges (52), where nitrate-phosphate imbalances caused by atmospheric depositions (53) may alter significantly the phytoplanktonic behavior and trophic status (54). However, pH is often the strongest segregator of community types (55–57). DNA was extracted, amplified and sequenced with standard methodology as previously described (4, 58), using a subset of 224 lake samples where both *Bacteria* and *Eukarya* were successfully sequenced. Briefly, high-speed multiplexed rRNA gene sequencing with the Illumina MiSeq System was carried out using the primers 515f and 806r (59) for the bacterial 16S V4 region and the primers 1391f and EukBr for the eukaryal 18S V9 region (60). Illumina paired-end raw data were processed and quality filtered using UPARSE (61), and zero-radius OTUs (zOTUs) were recovered with the UNOISE algorithm (62). To avoid a sequencing depth bias and to homogenize the presence/absence probabilities of zOTUs, we first rarefied both bacterial and eukaryotic communities to 5,000 sequences per sample. Then, we kept these zOTUs with a minimum occurrence of 10% (24 lakes). Each zOTU was annotated with its mean relative abundance in the data set, its dominant Environmental Ontology (EnvO) affinity in the case of bacteria through the SeqEnv pipeline (63), and its primary nutrient assimilation strategy in the case of eukaryotes (39), respectively. Taxonomy was then inferred using the SILVA 128 database through the SILVA-NGS online portal (64). Raw data are fully available under BioProject PRJNA413654.

In addition, we specifically selected environmental variables for the 224 samples that broadly represent the data set: pH, DOC, nitrates (NO_3_^–^), total phosphorus (TP), soluble reactive phosphorus, dissolved reactive silica, sulfate (SO_4_^2–^), water renewal time, and altitude as a joint proxy of temperature and UV radiation, as previously reported (4).

### Inferring potential interactions.

We used a modified version of the framework of Blois et al. (22) that assesses causes of species associations controlling for environmental and spatial factors. The method identifies aggregation, randomness, or segregation of pairs of species across a landscape and then estimates whether the deviations from randomness can be attributed to environmental filtering and/or dispersal limitation. While the original framework uses C scores to quantify the significance of species cooccurrences and relies on matrix randomizations, we opted for Veech’s probabilistic method (65) to carry out this task. This probabilistic method assumes that the probability of a species occurrence at a site is equal to its observed frequency among the set of sites considered, allowing for the calculation of the probability of cooccurrence between two species at a given number of sites under the assumption of independent distribution of the species. Therefore, the model produces an expected distribution of cooccurrences that can be used to assign significance to the actual observed cooccurrences. This method is analytically exact, and fast, and does not imply prior interactive structure. Also, it is based on presence-absence, which avoids problems derived from the compositional nature of microbial amplicon data, such as spurious correlations, low sensitivity due to relative frequencies, and alterations in its covariance structure (66, 67).

Initially, we calculated the probability that each pair of species cooccur more (aggregated pair) or less (segregated pair) than expected, given their occurrences. Then, for species pairs that deviated from the expectations (i.e., that were not random), we tried to identify the cause of these deviations, whether they are produced by environmental or spatial factors. Environmental factors were tested by one-way ANOVA of each environmental variable comparing allotypic sites (sites with only species A versus only species B) in segregated pairs or, in the case of aggregated pairs, sites with both species against empty sites. In the case of spatial factors, the null hypothesis tested with a one-way MANOVA of the coordinates of the sites is that the different types of sites are not spatially segregated. If this hypothesis is rejected, the cause of the observed pattern can be imputed to some form of dispersal limitation, at least partially. In summary, each pair of zOTUs can be assigned to one of the following results: random pairs, segregated pairs due to negative interactions or environmental filtering and/or dispersal limitation, and aggregated pairs due to positive interactions or environmental filtering and/or dispersal limitation (22).

### Network and node properties estimation.

We used the inferred positive and negative potential interactions to build a metacommunity network, where nodes (zOTUs) are linked with weighted links. These weights can be positive (cooccurrences) or negative (coexclusions), and their value was the logarithm of the cooccurrence/coexclusion *P* value obtained when testing the null hypothesis that cooccurrences/coexclusions emerge by chance (68). In practice, this means that weights were proportional to the order-of-magnitude difference between probabilities and the significance level.

We described network structure using standard statistical descriptors for complex networks implemented in iGraph (69). We analyzed network properties such as “diameter” (shortest-path length between the most distant nodes in the network), “average shortest-path length” (average number of steps it takes to get from one node of the network to another), and “clustering coefficient” defined as the average transitivity (the clustering of a node is defined as the ratio of existing links connecting its neighbors among each other to the maximum possible number of such links between neighbors; the network clustering coefficient is the average over the network of each node’s clustering). Modularity was calculated using the spinglass algorithm (70), which allows the definition of community clusters based on both positive and negative edges. The index reveals how significant network community clusters are, optimizing network partitions by including more positive edges within the modules and more negative edges between modules. Finally, we extracted the modules assigned by the spinglass algorithm for downstream analyses. The total number of edges connected to a given node is defined as “node degree.” By classifying zOTUs into network modules, we aimed at finding taxonomic differences in and between the modules and to assess keystone groups based on weighted node degree. When including the sum of the connection strength (weight) of every edge in the network, we refer to the node degree as the “weighted node degree” (71). The nodes that have a significantly larger degree, in comparison to other nodes in the network, are interpreted as hubs or keystones. These are nodes that can be interpreted as facilitator species, highly competitive species, or species subject to a high biotic pressure (i.e., predation).

### Statistics.

We used a chi-square test of independence to analyze contingency tables of the contribution of explanatory variables to taxonomic groups and also the contribution of taxonomy, EnvO (72), or nutrient assimilation strategies to different modules. We quantified the contribution in the percentage of the residuals to the total chi-square statistic to indicate particular associations that stand out, not having an effect on the differential interaction numbers included in the categories. To study the explanatory variables and their association with taxonomy at the genus level, we first grouped genera according to a clustering partition around k-medoids (PAM-K) and optimum average silhouette to estimate the optimum number of clusters, as implemented in the R package *fpc* (73, 74).

### Data availability.

The whole gene sequence data sets were deposited to the NCBI Sequence Read Archive and are available through BioProject PRJNA413654.

10.1128/msphere.00918-21.1 TEXT S1 Defining potential keystone taxa and guild components. Download Text S1, DOCX file, 0.01 MB. Copyright © 2022 Ontiveros et al. 2022 Ontiveros et al. https://creativecommons.org/licenses/by/4.0/ This content is distributed under the terms of the Creative Commons Attribution 4.0 International license.
