# Supplementary material for: Biological Microbial Interactions from Cooccurrence Networks in a High Mountain Lacustrine District
Source: mSphere. 2022 Jun 1;7(3):e00918-21. doi: 10.1128/msphere.00918-21 (PMC9241510; doi:10.1128/msphere.00918-21)
Supplement: FIG S1 [file msphere.00918-21-s0003.pdf]

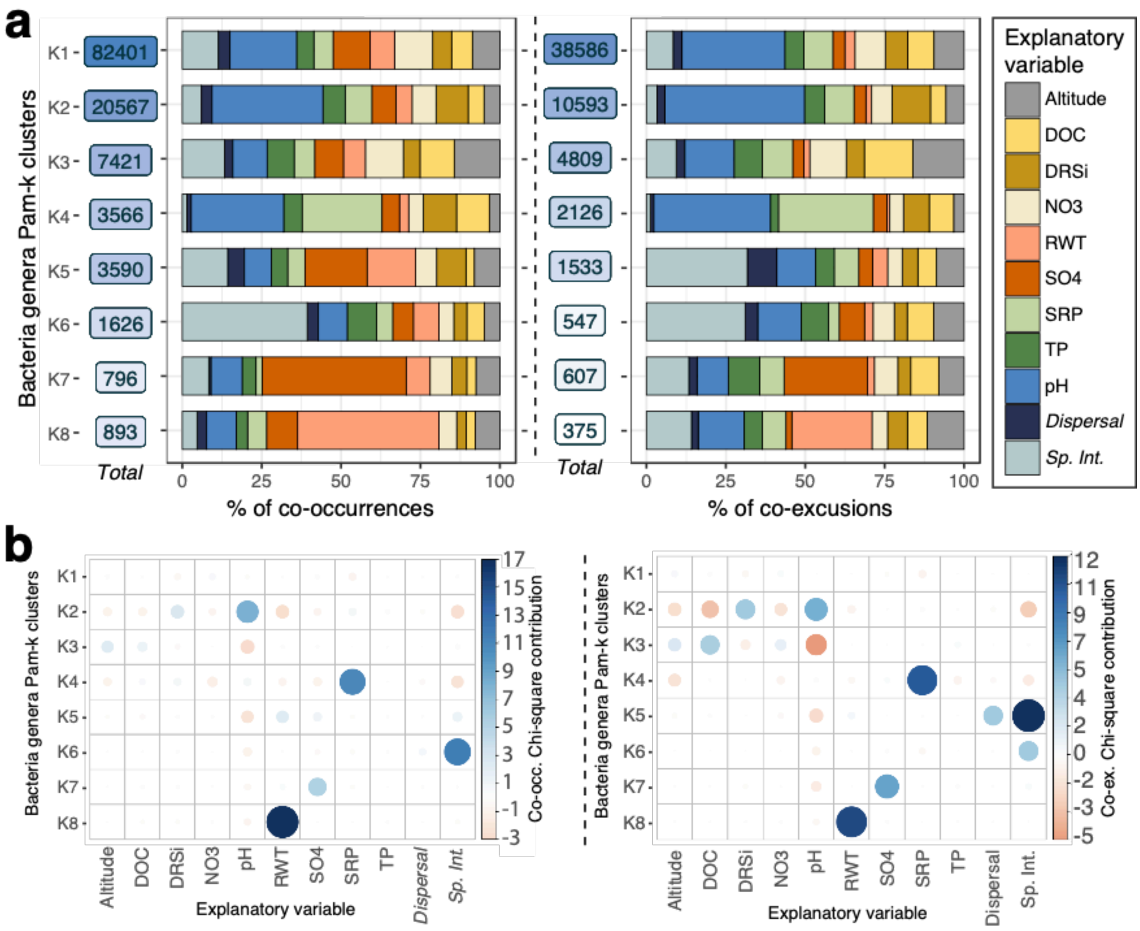

Figure S1. (**a**) Barplots displaying the proportions of variables (environment, dispersal or potential species interaction) that explain co-occurrences (left) and co-exclusions (right) across taxonomic bacterial genera, according to the results of the Blois et al., 2014 method. A single pair constitutes two nodes with the same or different taxonomy; hence, a pair could contribute to two different taxa. The barplot displays the groups of genera according to a partitioning around medoids clustering, relying on the proportion of explanatory variables over the pairs where each genus was participating. (**b**) Contribution in percentage of the Pearson residuals to the total Chi-square value of co-occurrences (left) and co-exclusions (right). We used a chi-square test of independence to analyze the contingency table of the relative contribution of explanatory variables to Pam k-medoids clusters. In co-occurrences, we observed a significant association between the categories of the two variables (p=0). The variables that contributed most to the association were pH (positively in K2), Sp.interaction (positively in K6), and RWT (positively in K8), and SRP (positively in K4). For co-exclusions, we also observed a significant association between the categories of the two variables (p=0). The variables that contributed most to the association were Sp.interaction (promoted in K5 and K6, and diminished in K2). PH contributed positively in K2, and negatively in K3. As for co-occurrences, RWT contributed positively in K8. SRP contributed positively in K4, and SO4 contributed positively in K7.
