# Supplementary material for: Biological Microbial Interactions from Cooccurrence Networks in a High Mountain Lacustrine District
Source: mSphere. 2022 Jun 1;7(3):e00918-21. doi: 10.1128/msphere.00918-21 (PMC9241510; doi:10.1128/msphere.00918-21)
Supplement: TABLE S1 [file msphere.00918-21-s0001.docx]

**Supplementary Table S1.** *List of bacterial genera grouped according to pam k-medoids clustering.*

**K1.** Aeromonas, Alpinimonas, Arcicella, Candidatus Hodgkinia, Candidatus Omnitrophus, Candidatus Planktoluna, Candidatus Planktophila, Chthoniobacter, CL500-29 marine group, CL500-3, Comamonas, Cryobacterium, Deefgea, Diaphorobacter, Duganella, Emticicia, endosymbionts2, Ferruginibacter, Filimonas, Flavobacterium, Flectobacillus, Fluviicola, GKS98 freshwater group, Janthinobacterium, Lautropia, Leptolyngbya, Limnohabitans, Luteolibacter, Lysinimonas, Massilia, MWH-Ta3, Mycobacterium, Novosphingobium, Opitutus, Paucimonas, Phreatobacter, Polymorphobacter, Polynucleobacter, Prochlorococcus, Pseudarcicella, Pseudomonas, Rhodovarius, Roseiflexus, Sediminibacterium, Skermanella, Sphingomonas, Synechococcus, Terrimicrobium, uncultured, Unclassified, Undibacterium, and Zavarzinia.

**K2.** Acidothermus, Albirhodobacter, Armatimonas, Brevundimonas, Delftia, Dinghuibacter, Haliscomenobacter, hgcI clade, Hydrogenophaga, Mycoplasma, Parasediminibacterium, Peredibacter, Polaromonas, Rhodobacter, Sandarakinorhabdus, Sinobacterium, Solitalea, Sphingorhabdus, Tetragenococcus, Vogesella, and Zavarzinella.

**K3.** Achromobacter, Acidocella, Acinetobacter, Aquabacterium, Arthrobacter, Candidatus Methylopumilus, Candidatus Symbiobacter, Cellvibrio, Cytophaga, Gemmatimonas, Herbaspirillum, Ideonella, Klebsiella, Methylotenera, and Ornatilinea, Ottowia, Paludibacter, Pantoea, Pedobacter, Phenylobacterium, Phormidium, Pirellula, PRD01a011B, Prosthecobacter, Rhodoferax, Roseococcus, and Serratia.

**K4.** Candidatus Aquirestis, Candidatus Captivus, Hirschia, Leadbetterella, Porphyrobacter, Rhizorhapis, and Sphingopyxis.

**K5.** Bacillus, Bacteriovorax, Bdellovibrio, Brachymonas, Carnobacterium, Caulobacter, Cloacibacterium, Inhella, Leeia, Legionella, Leptothrix, Methylobacterium, Methylorosula, ML602J-51, Ralstonia, Ramlibacter, Reyranella, Rhizobium, Sphaerotilus, and Sphingobium.

**K6.** Acidovorax, Candidatus Methylacidiphilum, Candidatus Xenohaliotis, Micrococcus, OM43-clade, Oxalobacter, Paludibaculum, Pelomonas, Pseudorhodoferax, and Stenotrophomonas.

**K7.** Aerococcus, Candidatus Rhodoluna, Chryseobacterium, Hymenobacter, and Staphylococcus.

**K8.** Chitinophaga, Curvibacter, Iodobacter, Microcystis, Noviherbaspirillum, Silvimonas, and Zymomonas.
