# Supplementary material for: Biological Microbial Interactions from Cooccurrence Networks in a High Mountain Lacustrine District
Source: mSphere. 2022 Jun 1;7(3):e00918-21. doi: 10.1128/msphere.00918-21 (PMC9241510; doi:10.1128/msphere.00918-21)
Supplement: FIG S2 [file msphere.00918-21-s0004.pdf]

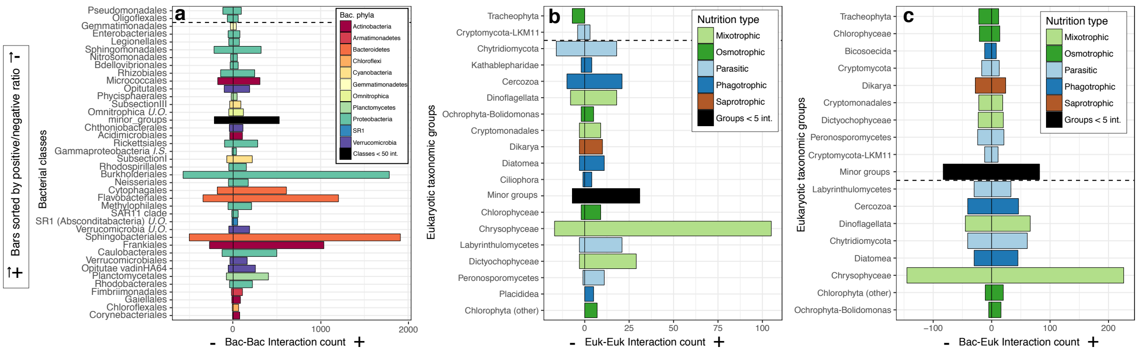

Figure S2. Summary of estimated interactions by taxonomic groups in Bacteria (**a**) and Eukarya (**b**), and interdomain (**c**). Raw counts, without weights. In terms of taxonomic groups, in Bacteria, members of Burkholderiales (Betaproteobacteria), Sphingobacteriales, Flavobacterales and Cytophagales (Bacteroidetes), Caulobacterales (Alphaproteobacteria), and Frankiales (Actinobacteria) held the most interactions (**Figure S2a**). Except the orders Pseudomonadales (Gammaproteobacteria) and Oligoflexales (Deltaproteobacteria), all orders had more positive than negative potential interactions. In the case of Eukarya (**Figure S2b**), most potential interactions were quantified in the dominant group Chrysophyceae, followed by Chytridiomycota, Dictyochophyceae, Cercozoa and Dinoflagellata. Only Cryptomycota had more negative than positive potential interactions, and Tracheophyta only was involved in negative pairs. Bacterial relationships with eukaryotes (**Figure S2c)** were concentrated in Chrysophyceae, and then in Dinoflagellata, Chytridiomycota and Cercozoa. Interestingly, 9 groups (out of 17) had more negative than positive interactions: Tracheophyta, Chlorophyceae, Bicosoecida, Cryptomycota, Dikarya, Cryptomonadales, Dictyochophyceae, Peronosporomycetes and Cryptomycota.
