# Supplementary material for: Biological Microbial Interactions from Cooccurrence Networks in a High Mountain Lacustrine District
Source: mSphere. 2022 Jun 1;7(3):e00918-21. doi: 10.1128/msphere.00918-21 (PMC9241510; doi:10.1128/msphere.00918-21)
Supplement: FIG S3 [file msphere.00918-21-s0005.pdf]

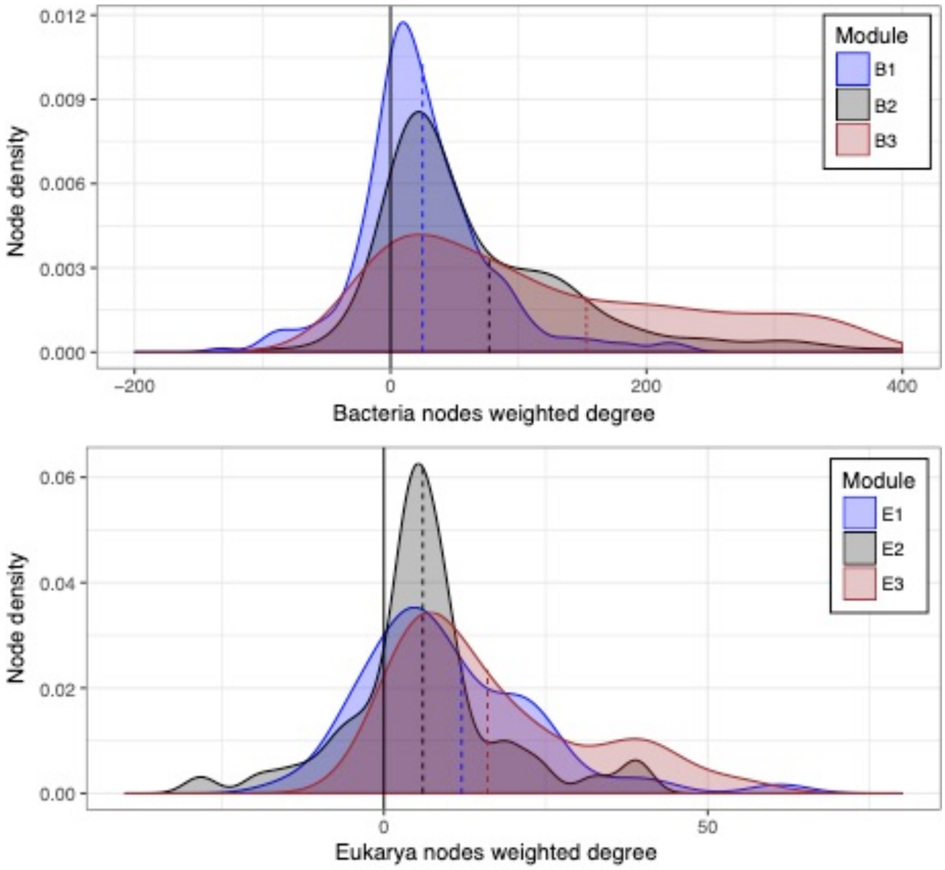

Figure S3. Density distribution of node weighted degree, displayed for the modules in the network of bacteria (a) and in the network of eukaryotes (b). The mean value of nodes from each module was drawn with a dashed line. Within bacteria, module B3 had the highest clustering, lowest average path length and highest hub values, in contrast to module B1. Within Eukarya, E1 had the highest clustering, and E2 had the lowest. Regarding node properties, we focus on the weighted node degree as one of the parameters with proven ecological significance. Weighted node degree follows a normal distribution closely centered at the 0 value, but skewed towards positive values. Density distribution means are different in the previously defined modules, all with positive means (B1 = 25, B2= 77, B3=153; E1=12, E2= 6, E3=16). In Bacteria, B1 was the module with the most nodes around small values and slightly skewed towards negative values, followed by B2, which had a fraction of nodes with notable weighted degree values, and B3 had the highest density of nodes above a weighted degree of 175. In the case of Eukarya, module E2 was the one with smallest values, E1 slightly skewed towards negative values and E3 slightly skewed towards positive values.
