# Supplementary material for: Biological Microbial Interactions from Cooccurrence Networks in a High Mountain Lacustrine District
Source: mSphere. 2022 Jun 1;7(3):e00918-21. doi: 10.1128/msphere.00918-21 (PMC9241510; doi:10.1128/msphere.00918-21)
Supplement: FIG S4 [file msphere.00918-21-s0006.pdf]

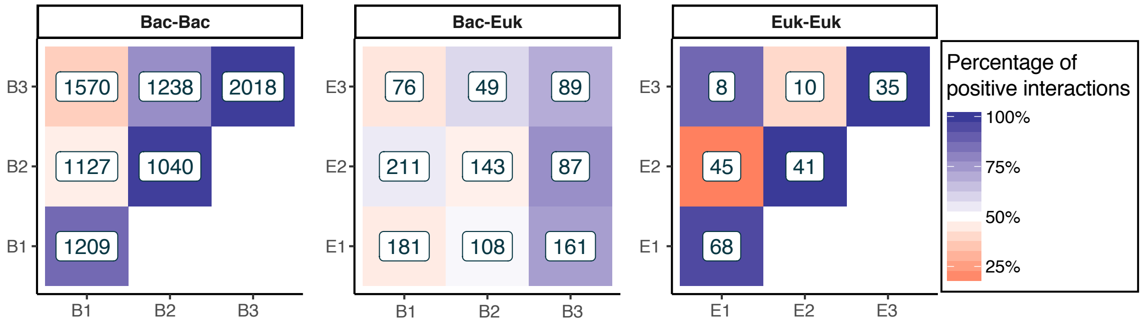

Figure S4. Number of edges between and within modules of the Bacteria network (B1, B2, B3), Eukarya network (E1, E2, E3), and inter-domain relationships based on domain-specific modules. Regarding the interrelationships between modules, in other words, edge distribution, in Bacteria the B1 module displays the most negative interactions towards B2 and B3. In Eukarya, the module with the most negative interactions is E2, towards modules E1 and E3. Between bacterial modules and eukaryotic modules, B1 showed more negative than positive interactions with E1 and E3, and B2 with E2.
