# Supplementary material for: Biological Microbial Interactions from Cooccurrence Networks in a High Mountain Lacustrine District
Source: mSphere. 2022 Jun 1;7(3):e00918-21. doi: 10.1128/msphere.00918-21 (PMC9241510; doi:10.1128/msphere.00918-21)
Supplement: FIG S5 [file msphere.00918-21-s0007.pdf]

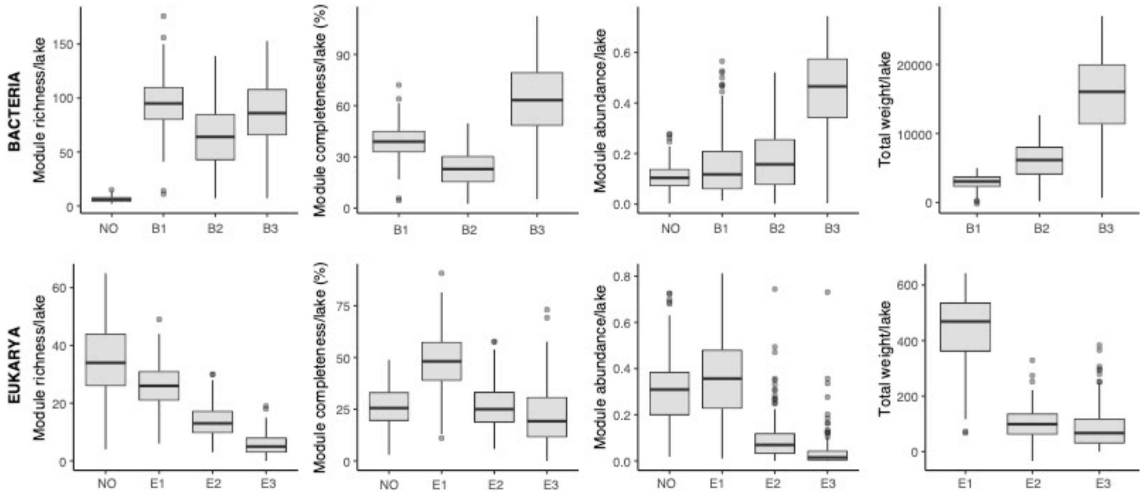


**Figure S5**. Local community quantification of network metrics: module richness/lake (number of zOTUs within a module that are present in each individual sample), module completeness/lake (proportion of nodes from each module that is present in each individual sample, regarding the total number of nodes of each module), module abundance/lake (the total relative abundance of the nodes corresponding to each module) and total node weight/lake (sum of node weighted degree by each module and each lake). In the bacterial modules, module B1 other than having the lowest weighted degrees, represented the lowest aggregated abundances per lake, but a similar high richness to module B3. Module B3 dominated with the highest abundances, highest degree and the highest completeness per lake, and B2 showing intermediate values and the lowest richness per lake. In the eukaryotic modules, E1 was the richest, the most complete and the most abundant per lakes, and also had the highest aggregated weighted degree. In the other hand E3 showed the lowest richness, completeness, abundance and weighted degrees per lake.
