# Supplementary material for: Biological Microbial Interactions from Cooccurrence Networks in a High Mountain Lacustrine District
Source: mSphere. 2022 Jun 1;7(3):e00918-21. doi: 10.1128/msphere.00918-21 (PMC9241510; doi:10.1128/msphere.00918-21)
Supplement: TEXT S1 [file msphere.00918-21-s0002.docx]

**Supplementary Table S2**

*Defining potential keystone taxa and guild components*

To define keystone taxa we evaluated the mean weighted degree of nodes assigned to each bacterial order, including both positive and negative edges. Hence, a positive value may include also nodes with negative degrees although not explicitly indicated. Also, because organisms from the same taxonomic group may not have the same behavior in different modules, we have differentiated nodes according to their assigned modules. Overall, we observed three possibilities: one, taxa behaving similarly in the modules where these are present, as we observe in the Alphaproteobacteria group, with orders having mid-high abundances and a trend towards positive interactions; similarly happening in the Bacteroidetes groups (Cytophagales, Flavobacterales and Sphingobacteriales). A second possibility are module-exclusive groups, or groups that do not appear in a module. A good example are the organisms within Firmicutes, relevant in module B1 but absent or nearly absent in modules B2 and B3. A third possibility is the observation of different behaviors of the same orders in all the modules. A good example is the Enterobacteriales order, with a negative aggregated weighted degree in the B1 module, positive in the B2, and absent in B3.

This strategy, together with a quantile-based assessment of node mean abundances, can help discern keystone taxa in the network modules for each domain. Integrating abundances gives an additional layer of information, since their node relative abundances do not correlate with the weighted degree, hence with the interaction potential of different taxa. In the bacterial module B1 the orders with the highest weighted degrees were a low abundant order of Cyanobacteria (Subsection I), Myxococcales, Erysipelotrichales (Firmicutes), or the high abundant Verrucomicrobiales; while the orders with the lowest –negative- degrees were Micrococcales genera (*Lysinimonas*, *Alpinimonas* and *Athrobacter*), Lactobacillales genus (*Aerococcus*), high abundant Enterobacteriales genera (*Serratia*, *Pantoea*), Legionellales genus (*Legionella*) or Xanthomonadales. The bacterial module B2 had mostly positive weighted degrees, with the highest degrees found in Fimbriimonadales (Armatimonadetes), Cytophagales, or the low abundant Legionellales. Finally, bacterial module B3 had also many positive interactions and the highest weighted degrees, with the outstanding values of the OM23 Clade (Methylophilales, in Betaproteobacteria), the biofilm-forming Caulobacterales (Alphaproteobacteria), Gaiellales (Actinobacteria), and all the Verrucomicrobia groups.
